# Pseudohypoaldosteronism Type 1b in a Saudi Female Infant Due to Homozygous Variant Gene Mutation in SCNN1A: A Case Report

**DOI:** 10.7759/cureus.67165

**Published:** 2024-08-19

**Authors:** Ali Alquraishi, Abdullah Alshahrany, Badriah G Alasmari, Ashwaq Hommadi, Mohammed Alomari

**Affiliations:** 1 Pediatric Endocrinology, Armed Forces Hospital Southern Region, Khamis Mushait, SAU; 2 Pediatrics, Armed Forces Hospital Southern Region, Khamis Mushait, SAU

**Keywords:** scnn1a, metabolic acidosis, hyponatremia, hyperkalemia, pha1b, pseudohypoaldosteronism

## Abstract

Pseudohypoaldosteronism type 1 (PHA1) is a rare, heterogeneous group of disorders characterized by resistance to aldosterone action. We report the case of a 17-day-old Saudi female infant who presented on the third day of life with persistent hyperkalemia, hyponatremia, and metabolic acidosis. Initial evaluation for congenital adrenal hyperplasia was unremarkable. Genetic testing confirmed a novel homozygous variant (c.1522C>T p.(Arg508) chr 12:6458147 in SCNN1 A) in the *SCNN1A* gene, consistent with the diagnosis of PHA1B, a genetically confirmed subtype of PHA1. Prompt recognition and management of electrolyte disturbances are crucial in these neonates to prevent life-threatening complications.

## Introduction

Pseudohypoaldosteronism type 1 (PHA1) is an uncommon inherited disorder resulting from end-organ resistance to the biological activity of aldosterone. There are two main forms of PHA1: renal and generalized [1,2]. Due to the rarity of this condition, the majority of published literature comprises scattered case reports or small case series. Herein, we report the case of a Saudi female infant with PHA1B, a genetically confirmed subtype of PHA1.

## Case presentation

A 17-day-old Saudi female infant, the product of a 39-week gestation born to consanguineous parents, was referred to our hospital with persistent hyperkalemia (potassium=9 mmol/L) and hyponatremia (sodium=126 mmol/L). Her symptoms started on the third day of life with vomiting, poor feeding, and decreased activity. She required mechanical ventilation and peritoneal dialysis at the referring hospital before transfer to our center for further management and evaluation by a pediatric endocrinologist.

On presentation, the infant appeared severely ill and dehydrated, with pale skin and poor perfusion (capillary refill time=3 s). Her genitalia were normal, and there was no hyperpigmentation. Aggressive management of the electrolyte imbalance and dehydration was initiated, including intravenous normal saline, sodium bicarbonate, calcium gluconate, salbutamol nebulizers, and a glucose-insulin infusion.

As seen in Table 1, hormonal studies showed normal baseline early-morning cortisol (220 nmol/L), 17-hydroxyprogesterone (5.6 ng/mL), and adrenocorticotropic hormone (ACTH, 21.9 pg/mL) levels. However, her aldosterone level was elevated (100 ng/dL). Her ACTH stimulation test was normal, and an abdominal ultrasound revealed normal adrenal glands. These findings helped to rule out congenital adrenal hyperplasia.

**Table 1:** Laboratory test results for the patient ACTH: Adrenocorticotropic hormone

Laboratory Test	Patient’s Value	Normal Range (Neonatal)
Potassium (K^+^)	9mmol/L	3.5–5.5 mmol/L
Sodium (Na^+^)	126 mmol/L	135–145 mmol/L
Cortisol	220 nmol/L	140–690 nmol/L
17-hydroxyprogesterone	5.6 ng/mL	0.7–9.0 ng/mL
ACTH	21.9 ng/mL	10–46 pg/mL
Aldosterone	100 ng/dL	10–60 ng/dL

Whole exome sequencing identified a novel homozygous variant (c.1522C>T p.(Arg508) chr 12:6458147 in SCNN1 A) in the SCNN1A gene, confirming the diagnosis of PHA1B. This variant is found in 0.0033% of the overall population and has not been previously reported in the literature.

The patient’s condition was successfully stabilized in the pediatric intensive care unit. Her serum sodium and potassium levels were normalized. During inpatient admission, the patient required administration of the following crucial medications: 3% sodium chloride solution, sodium bicarbonate, calcium resonium, and fludrocortisone. The patient exhibited an excellent therapeutic response without the development of any complications. A nasogastric tube was required to facilitate enteral feeding and provision of oral supplements.

Following one month of inpatient management, the patient was discharged and returned home in an excellent state of health. The discharge medication regimen consisted of 3% sodium chloride solution 7 mL administered orally via the nasogastric tube every 4 h, calcium resonium 1 gram orally twice daily, and fludrocortisone 0.3 mg orally once daily. An outpatient endocrinology clinic follow-up appointment was scheduled for six weeks post-discharge.

At the endocrinology clinic visit, the patient’s overall clinical condition was found to be stable, with normal vital signs and serum electrolyte levels. The patient continued to attend regular follow-up appointments in the endocrinology clinic every 2-3 months until 2.5 years of age. During this period, the patient’s weight increased from the initial 3 kg at first admission to 8.7 kg at the last clinic visit. The patient was subsequently lost to follow-up and did not continue to come to our clinic regularly. Her current age is five years old.

Since discharge from the initial hospitalization, the patient was intermittently brought to the emergency department for episodes of vomiting, intolerance to oral feeding and supplements, and febrile illnesses. These intercurrent events were managed with intravenous fluid resuscitation, intravenous antibiotic therapy, pantoprazole, and targeted correction of any hyperkalemia or hyponatremia using dextrose 5% in normal saline, calcium gluconate, Ventolin nebulization, insulin/dextrose infusion, and intravenous sodium supplementation. The patient responded rapidly to this comprehensive management and was consistently discharged in an excellent state of health.

Overall, the patient’s response to the comprehensive treatment regimen was outstanding, and the clinical outcome was generally very favorable. However, significant challenges remained, including failure to thrive, intolerance to oral supplements, and recurrent electrolyte disturbances precipitated by intercurrent illnesses, necessitating close clinical monitoring and follow-up care.

## Discussion

PHA1 is a rare genetic disorder characterized by end-organ resistance to aldosterone, leading to severe electrolyte imbalances that can be life-threatening if not promptly recognized and managed [1,2]. Hyperkalemia, a hallmark of PHA1, is a serious metabolic derangement that can result in fatal cardiac arrhythmias [3]. Moreover, hyperkalemia has been widely documented as a major contributing factor in sudden infant death syndrome [4]. Timely identification and aggressive management of hyperkalemia are crucial life-saving therapeutic strategies.

An amalgamation of hyponatremia, hyperkalemia, and metabolic acidosis may indicate underlying adrenal insufficiency, and therapy with corticosteroids can provide a tangible response [5]. However, in cases where the response to corticosteroid therapy is not beneficial or the clinical presentation is unusual, peripheral resistance to aldosterone should be highly considered [6,7].

PHA1 is a clinical disorder characterized by obvious insensitivity of the renal tubules to aldosterone activity. PHA1 comes in two forms: renal and generalized [1,2]. These forms differ in their genetic basis and clinical manifestations.

The renal form of PHA1 is inherited in an autosomal-dominant manner. The underlying genetic alteration involves a heterozygous mutation in the mineralocorticoid receptor gene, NR3C2, located on chromosome 4 [1,2]. Clinically, newborns with the renal form of PHA1 may be asymptomatic, and the condition may be diagnosed by detecting high aldosterone serum concentrations in patients with salt-wasting renal diseases. However, the condition often manifests with vomiting, dehydration, and failure to thrive during early infancy. Hyperkalemia is generally not severe, and it can be effectively managed with sodium supplementation. The renal form of PHA1 tends to improve with age, and the prognosis is generally favorable. Nonetheless, significant mortality has been reported in some cases [8].

The generalized form of PHA1 is inherited in an autosomal-recessive manner. The underlying genetic alterations involve mutations in the epithelial sodium channel subunit genes SCNN1A (chromosome 12) or SCNN1B and SCNN1G (chromosome 16) [1,2]. Newborns with the generalized form typically experience dramatic electrolyte imbalances, severe dehydration, and potentially cardiovascular failure. Unlike the renal form, the generalized form is characterized by aldosterone resistance affecting not only the renal tubules but also other body organs. Common features include chronic and progressive recurrent pulmonary infections, wheezing, sodium wasting through salivary glands and skin sweat, and recurrent skin infections. The clinical presentation of the generalized form may resemble and be misinterpreted as cystic fibrosis. The prognosis of the generalized form is very poor, and newborns often suffer the consequences of recurrent life-threatening salt-wasting incidents.

Prompt and accurate diagnosis of PHA1 is crucial. The laboratory profile typically includes increased aldosterone, renin, serum potassium, and urinary sodium levels, as well as decreased serum sodium and urinary potassium levels, which establishes the diagnosis of PHA1 from a clinical perspective [1,2,6,7]. Genetic analysis is required to differentiate between the renal and generalized forms of PHA1 and confirm the diagnosis.

Differential diagnosis of PHA1 includes a wide array of adrenal diseases, such as adrenal hypoplasia and congenital adrenal hyperplasia [2]. Therefore, laboratory testing of serum cortisol, serum 17-hydroxyprogesterone, and urinary steroid concentrations is essential. Additionally, a temporary and transient form of PHA, known as PHA type 3, can occur due to underlying renal congenital anomalies or ongoing urinary tract infections, warranting ultrasound studies of the renal system and urine culture [1,9,10].

Management of PHA1 is largely guided by the type of form involved (i.e., renal or generalized).

Management of the renal form often involves adequate rehydration, electrolyte correction, potassium exchange resin, and sodium supplementation. These requirements typically decrease with age, usually beyond two years of age.

The generalized form of PHA1 is often associated with worse outcomes and requires more aggressive fluid-electrolyte supplementation. Enteral feeding and intravenous routes may become necessary during the course of therapy. Unfortunately, treatment for the generalized form is typically lifelong. In patients with cardiovascular collapse, indomethacin and thiazide diuretics may be administered.

## Conclusions

PHA1, especially the autosomal recessive generalized form, is a potentially life-threatening disorder in neonates. Early diagnosis with a high index of suspicion and prompt management are essential. In such cases, congenital adrenal hyperplasia should be ruled out, and genetic studies can provide accurate diagnosis, facilitate genetic counseling, and enhance understanding of the pathogenesis of PHA1.
